# Decision-Making Time Analysis for Assessing Processing Speed in Athletes during Motor Reaction Tasks

**DOI:** 10.3390/sports12060151

**Published:** 2024-05-29

**Authors:** Leonardo Ariel Cano, Gonzalo Daniel Gerez, María Soledad García, Ana Lía Albarracín, Fernando Daniel Farfán, Eduardo Fernández-Jover

**Affiliations:** 1 Neuroscience and Applied Technologies Laboratory (LINTEC), Bioengineering Department, Faculty of Exact Sciences and Technology (FACET), National University of Tucuman (UNT), Superior Institute of Biological Research (INSIBIO), National Scientific and Technical Research Council (CONICET), Av. Independencia 1800, San Miguel de Tucumán 4000, Argentina; 2 Faculty of Physical Education (FACDEF), National University of Tucuman (UNT), Av. Benjamin Araoz 750, San Miguel de Tucuman 4000, Argentina; 3 Institute of Bioengineering, Universidad Miguel Hernández of Elche, 03202 Elche, Spain; 4 Research Networking Center in Bioengineering, Biomaterials and Nanomedicine (CIBER-BBN), 28029 Madrid, Spain

**Keywords:** reaction time, decision-making, electromyography, sport performance, handedness

## Abstract

Reaction time (RT) is a widely used measure for testing physical performance in motor tasks. This study focused on assessing the processing speed in athletes. Twenty-five healthy volunteers were assigned to the control (n = 16) or athletes groups (n = 9). They were evaluated during motor reaction tasks based on visual stimuli and three difficulty conditions. Physiological measures were obtained from motion capture and electromyography recordings of several muscles. Two RT phases, decision-making (DMK) and electromechanical delay (EMD), were used to analyze the processing speed. The results show significant RT differences between groups. The athletes were ~30% faster compared to the control group. Despite the fact that all participants were right-handed, RT did not show any differences between hands performances in any group. However, DMK time revealed significant differences between the hands. Controls showed a longer DMK time for the right-hand election, ~20% more than the left, while athletes showed no such disparity. These findings reveal that quantifying the decision-making component of reaction time is crucial to assessing processing speed in sport. This approach could facilitate the monitoring of adaptations in both motor–cognitive and neuromuscular processes. The theoretical implications presented in this study offer perspectives on handedness research.

## 1. Introduction

Understanding the neural processes related to decision-making has been a consistent challenge for scientific efforts. Thus, reaction time (RT) in simple tasks has been proposed as a metric of processing speed [1], operationally understood as the time required to complete the processes (central and peripheral) to make a response [2]. Such processes involved during RT range from scanning the environment to identifying the object of interest, deciding the response and its features, and evoking the motor program that will initiate the planned action. It is possible to define this set of processes as decision-making (DMK) [3].

Once the motor command is executed at the central level, action potentials can be detected in the muscle at the peripheral level, initiating the neuromuscular response. Surface electromyography (EMG) allows for the detection of the onset of motor unit recruitment, a process that requires a certain amount of time until mechanical transduction is manifested. This time is known as electromechanical delay (EMD) [4] and depends on electrochemical and neurophysiological components [5]. In this regard, employing EMG makes it possible to differentiate between the DMK and EMD phases within RT [6].

In motor tasks, it has been demonstrated that several factors can modulate RT, such as context, prior experience, stimulus complexity, compatibility with the response, number of possible responses, or accuracy required [2]. In particular, in reaching tasks, RT can be influenced by handedness. In this topic, the literature presents contradictory evidence. For instance, Dexheimer et al. [7] and Rabbitt [8] found shorter RTs when the task was performed with the dominant hand, whereas Velay [9] observed shorter RTs when right-handed individuals performed the task with their left hand. Furthermore, Barthélémy and Boulinguez [10] demonstrated that tasks performed with the left hand resulted in shorter RTs. In the sports field, athletes are required to use limbs for various actions, both symmetrical and asymmetrical, such as throwing, striking, grabbing or intercepting, with or without an object in between. In this sense, research on laterality becomes essential for assessing motor execution performances, in both dominant and non-dominant limbs. For instance, Badau et al. [11] reported that athletes from boxing, gymnastics, judo, karate, taekwondo, and wrestling exhibited higher performance in simple RT tasks with their left hand compared to their right hand, regardless of handedness. However, they showed that the dominant hand demonstrated greater efficiency in more complex tasks. The relationship between handedness and performance in processing speed remains unclear.

Shorter RT was associated with improved performance indicators in both physical capacity and sports skills [12,13,14,15,16]. RT is a key factor in sports performance, influenced by the ability to analyze and decide, as well as the speed of motor responses [17]. It has been stated that RT can be improved through the implementation of specific training programs [14]. In this line, athletes demonstrate higher performance in RT compared to control groups in tasks involving simple reaction [17,18,19,20]. Furthermore, RT has been used for revealing differences between athletes in closed- and open-skill sports. For instance, Nuri et al. [12] showed that athletes have enhanced sensory-cognitive abilities within their respective sport domains. Volleyball players demonstrate superior anticipation skills in ball timing tasks compared to sprinters. Conversely, sprinters exhibit faster RT to auditory stimuli. While this difference could be related to sport specificity, Vaeyens et al. [21] postulated that anticipatory skill plays an important role in successful DMK, particularly in team ball sports such as volleyball, soccer, basketball and handball, in which players must be aware of the activities and positions of multiple players simultaneously. In this sense, it has been suggested [22,23] that RT under a go/no-go task could be used as an index of expertise for sport-specific DMK. Kida et al. [23] reported simple RT did not vary significantly across levels of sports experience among baseball players. In contrast, baseball players exhibited significantly shorter go/no-go RTs compared to tennis players and controls. Moreover, among baseball players, those with higher skill levels demonstrated significantly shorter go/no-go RTs compared to less skilled ones. In relation to long-term sports adaptations, in a longitudinal study [23] over two years of hitting practice, the players improved the go/no-go RT, while the simple RT remained constant. This evidence leads to exploring whether the differences are related to motor–cognitive adaptations, specific muscle adaptations, or both.

Regarding the use of RT as a measure of processing speed, we propose to identify temporal differences within the RT by exploring its components (i.e., DMK and EMD). We hypothesized that, if the differences are due to central or peripheral processes, they could be related to DMK or EMD components, respectively. To this end, motor reaction tasks were designed under a go/no-go protocol with visual stimuli among different difficulty conditions, during which motion capture and EMG activity from several muscles were recorded to determine the phases of analysis (i.e., DMK, EMD). The aim of this study was to assess processing speed in athletes and controls by examining inter-group, inter-conditions, and inter-limb differences. This study could have potential future implications for researching tracking adaptations during physical–motor training and assessing handedness.

## 2. Materials and Methods

### 2.1. Participants

Twenty-five healthy volunteers took part in a motor reaction study involving visual stimuli. A non-probabilistic sampling of volunteers was conducted among university students, reflecting a diverse pool of participants eager to contribute to the study. All participants self-reported as right-handed, normal or corrected-to-normal vision, and declared no history of neurological or locomotor disorders. The inclusion of both male and female participants was a given, and our research design intentionally sought to reflect this diversity to ensure the generalizability of our findings. The control group (CON) consisted of 16 healthy participants (9 males, 7 females) with an average age of 26.4 ± 4.9 years; height 171.5 ± 9.4 cm; weight 62.9 ± 10.8 kg; Body Mass Index 21.2 ± 2.2 kg/m^2^. The inclusion criteria for CON were do not participate in organized sport, were physically active according to ACSM’s guidelines [24], and were not limited in their motor ability to participate in the study. The athletes group (ATH) comprised 9 participants (5 males, 4 females) who were selected from the volunteers and engaged in sports requiring the asymmetrical use of the upper limbs, such as karate, basketball, and tennis, with an average age of 25.2 ± 6.7 years; height 173.6 ± 11.9 cm; weight 66.1 ± 11.8 kg; Body Mass Index 21.8 ± 1.8 kg/m^2^. The inclusion criteria for ATH were currently practicing their sports, training at least three times per week, and consistent participation in national-level competitions over the past five years. All participants were instructed to arrive at the laboratory in a rested state, avoiding any strenuous activity in the previous 24 h. The participants were asked to avoid any food or drink containing caffeine for at least 12 h preceding the session. The study was conducted by following the ethical guidelines established in the Declaration of Helsinki and approved by the ethics committee of the Miguel Hernandez University from Elche, Spain (Reference: IB.EFJ.04.21). All participants were previously instructed about the tasks and gave written informed consent.

### 2.2. Experimental Design

**Figure 1:**
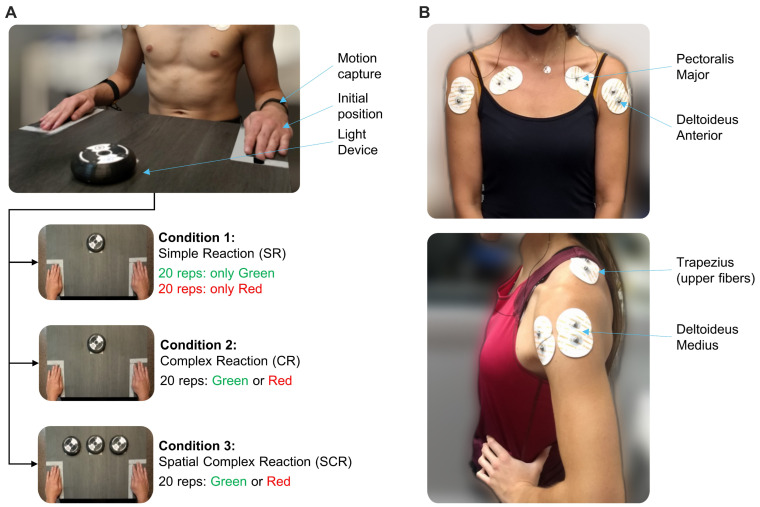
Experimental setup. (**A**) Photograph depicting the initial position of the participant. The tapes are attached to both forearms for detecting either hand movement. The participant was instructed to move their right hand towards the device when the green light turns on, and the red light indicated movement of the left hand. The subfigures below illustrate the motor tasks of three experimental conditions. (**B**) The EMG sensors’ locations for collecting muscle activity from *Pectoralis Major* (*pars clavicularis*), *Deltoideus Anterior*, *Trapezius* (*upper fibers*), and *Deltoideus Medius*.

The experimental protocol was based on a previous study [25]. The procedure took place in a quiet room designed for neuroscience research, where the room temperature was maintained within the range of 20 to 24 °C. The room was equipped with amenities for noise isolation and minimizing distractions. The experiment consisted of sitting the participant in front of a table with their hands located in a predefined position (Figure 1A). A device (reactimeter) was placed on the table and in front of the participant. The reactimeter emits programmed lights following a predefined protocol. The device emits one-color light (red or green) and includes a motion sensor. When the participant places their hand on the reactimeter, the light turns off; then, it is possible to measure the time lapse between the light being turned on and turned off. The participant was instructed to associate the green color stimulus with right-hand movement, and the red color with left-hand movement. Three experimental conditions were established for motor tasks, and the sequence of the three conditions was consistent across all participants: (1) “Simple Reaction” (SR) condition—The device emitted only one predetermined color, so the participant knew in advance that the hand should be moved before the light appeared. The initial light color presentation was randomized. Thus, half of the participants started with the green light and the other half with the red light. The participant performed twenty repetitions with each hand. (2) “Complex Reaction” (CR) condition—The device randomly emitted green or red light, so the participant had to make a decision before moving any hand. Twenty repetitions were performed in this condition. (3) “Spatial Complex Reaction” (SCR) condition—Three devices were employed simultaneously, and the green or red light could appear on any one of them, with the participant required to decide which hand to move, and then to plan the movement direction after processing the stimulus. The distance between the devices was set at 15 cm, ensuring that all were within the participant’s field of view without the need to move their head. Twenty repetitions were performed in this condition. The absence of a learning effect related to the repetitive motor task was verified. Supporting data, statistical analysis, and principal outcomes, along with extensive explanations, can be found in the Supplementary Material.

For all conditions, the minimum time between repetitions had to be 6 s; after that, the stimulus was spontaneously delivered. Participants were unaware of this information and were warned to remain attentive. The time for resting between series was 2 min. This permitted participants to rest from the sustained attentional demand (approximately 2 min) of each task and minimized mental dispersion from potential distractions. Consequently, participants’ attention on the entire experiment did not exceed 15 min in total. In order to avoid extra movements but the hands, the device was located 30 cm from the hands’ initial position. Additionally, participants were instructed to keep their gaze fixed on the central device.

Participants received specific instructions regarding the task. They were instructed to remain seated with their hands resting in designated positions and their muscles relaxed. In the first condition, SR, they were informed that a red/green light will appear on the device in front of them, and they should move the corresponding hand towards the device in response to this stimulus. For conditions involving decisions with more than one option (i.e., CR and SCR), they were instructed to take time to decide which hand to move. They were advised that they would perform twenty repetitions, and that stimuli would appear at random times. In the CR condition, they were informed that stimuli could appear in red or green randomly. In the SCR condition, they were told that the stimulus would only appear on one of the three devices, and the color would be random. Before the first test, participants performed a trial to test their comfort. The main test started when the participant was completely familiar with the test procedure. At the beginning of each task, the researcher provided a preparatory and start signal. During data collection, silence was prioritized to allow the participant’s complete concentration. At the end of each task, the researcher programmed the next task during the 2 min rest period.

### 2.3. Instruments

**Figure 2:**
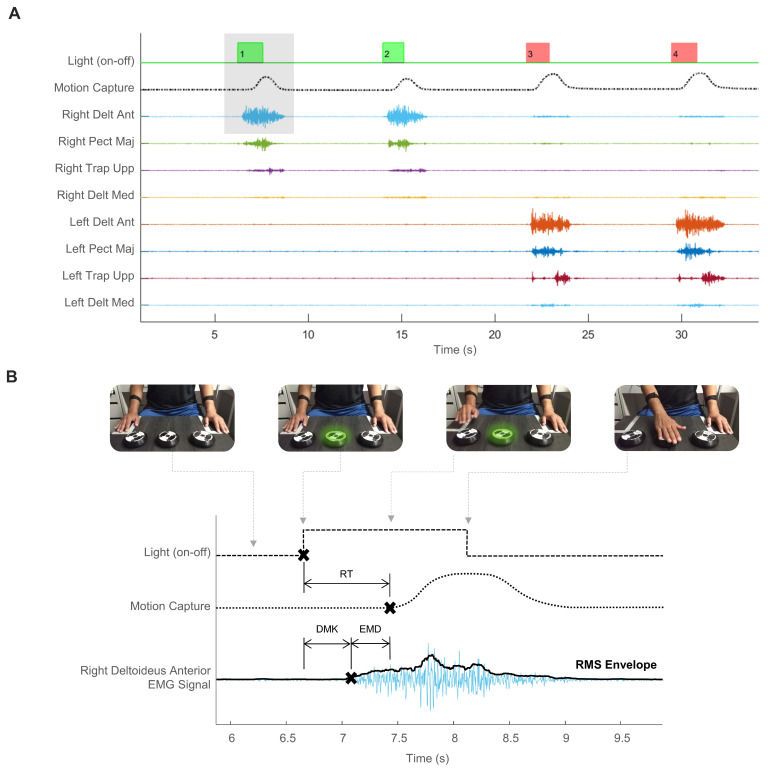
Diagram showing all synchronized time-series for measuring reaction time. (**A**) Partial data depicting four repetitions of complex reaction task over time. The upper line shows the reactimeter signal, binary on–off data are marked by green and red, depending on the light stimulus shown. The second line shows motion capture when either hand was moved. These data are an analog time-series scaled in millimeters derived from digital signals collected with a linear position transducer. The movement of either hand is detected when there is a change in the steady state, transitioning from a flat line to an increasing amplitude following the presentation of a visual stimulus. The next lines depict the normalized EMG data from eight muscles. (**B**) Zoom-in of the light gray rectangle in panel A. Reaction time (RT) is the sum of decision-making (DMK) and electromechanical delay (EMD). The crosses (×) indicate the onset of light, movement, and muscle contraction. The photographs represent four instants of the motor task: waiting for stimulus, device light turning on, starting right-hand movement after the green light turned on, and reaching the target with right hand, then the device light turned off.

Superficial EMG recordings were acquired by an RHA2000-series (Intan, Los Angeles, CA, USA) acquisition system (16-channel amplifier and 25 kHz sampling rate). Muscle activity data were collected using Ag/AgCl button electrodes (Dormo, Barcelona, Spain); skin surface preparation and electrode placements were performed following SENIAM guidelines. While SENIAM does not provide indications for electrode placement for the *m. Pectoralis Major*, we collected EMG activity from the *pars clavicularis* following the recommendations of previous studies [26,27] (Figure 1B). To measure the time, we used a system designed and manufactured in our laboratory (LINTEC, San Miguel de Tucuman, Argentina). It consists of a control center programmed for sending the signal via WiFi to the device to turn on/off the light. The reactimeter was developed to fulfill specific requirements of the protocol that were not supported by commercial systems. For example, it incorporates an external synchronization system with other devices, enabling real-time data acquisition without delay, and offers versatility in programming stimulus delivery (number of devices, colors, timing, and pauses). A linear position transducer with 1 kHz sampling rate (WinLaborat, Buenos Aires, Argentina) was attached to both forearms by adjustable tapes in order to detect hand movements (Figure 1A). The data from the position transducer and the reactimeter were acquired using the Intan board’s auxiliary digital channels. Cables connected from both instruments to the Intan facilitated this acquisition. The digital channels recorded data from these two instruments as time series, operating in parallel with the EMG recording. This synchronization ensured continuous alignment of all three types of signals, thereby preventing any lag or delay that could compromise data accuracy. Extensive details of the instrumental setup can be found in a previous study [25]. The three types of signal are depicted in Figure 2A.

### 2.4. Agonist Muscle Identification

Data from eight muscles (four per side) were collected: *Trapezius* (*upper fibers*), *Deltoideus Anterior*, *Deltoideus Medius*, and *Pectoralis Major* (*pars clavicularis*). To determine muscle participation, we relied on the triphasic EMG pattern [28,29]. As Correia et al. [6] explained, this pattern consists of three sequential bursts of EMG activity interspersed by periods of EMG silence, and it is mostly observed in fast movements. The first burst corresponds to the agonist that initiates the planned movement. Then, there is an increase in antagonist muscle activity to provide a braking force when approaching the target. Finally, another smaller agonist burst fine-tunes the final movement position. Among the four muscles in each arm, the *Deltoideus Anterior* was identified as the agonist muscle in every condition. This identification was carried out by following the established methodology outlined in prior work [30]. This procedure consisted in normalizing the EMG signal with the interval max value within the reaction time (RT), for each separated repetition. Second, we detected the envelopes with the root mean square (RMS) of every repetition. Third, the onset of the effective contraction was determined as the envelope amplitude exceeding three standard deviations over the basal state. The same criteria were applied for determining hand movement onset (see the crosses × in Figure 2B), corresponding to the end-point of RT.

### 2.5. Signal Processing and Phase Detection

All further analyses were performed offline with Matlab (MathWorks, Natick, MA, USA), version 2020b. While the EMG signals were acquired at a sampling frequency of 25 kHz, they were downsampled to 1 kHz using Matlab. The motion data from the position transducer were analyzed using classical kinematics formulations for obtaining displacement data. The position transducer comprises a rotational encoder providing three digital signals through distinct channels: one signal is movement detection; another is direction in one; and the third is direction in the other. Using these data, we developed a Matlab algorithm to convert these signals into an analog time-series. By adhering to the manufacturer’s specifications (i.e., encoder diameter, sampling frequency, and distance per pulses), we derived an analog time-series scaled in millimeters (Figure 2A). The reactimeter data were kept as binary on–off data. Specific events during the task (i.e., light turning on, muscle contraction onset, and hand movement onset) were used to separate the phases within RT (Figure 2B). As we have mentioned above, *Deltoideus Anterior* was identified as the agonist muscle. Determining the onset of the agonist muscle activity is crucial to splitting RT into the decision-making (DMK) phase and electromechanical delay (EMD).

### 2.6. Time Analysis

We determined the durations of the DMK and EMD phases using the following calculations: RT corresponds to the interval between light turning on and the onset of hand movement. DMK_TIME_ corresponds to the interval between light turning on and the onset of muscle contraction, representing the duration of the decision-making process. EMD_TIME_ denotes the period between the onset of muscle contraction and the initiation of hand movement, reflecting the time taken by the agonist muscle to initiate the movement. An offline inspection looking at all data was conducted to detect execution errors. The criterion for determining which repetitions should be discarded was based on the hand movement data captured by the position transducer; for example, if movement was detected before the stimulus appeared, or if there was incorrect moved-hand relative to the presented color, or if there was simultaneous movement of both hands. Repetitions with errors were excluded from the analysis. In the SR condition, 1.3% of the repetitions for the right hand and 0.3% for the left hand were discarded. In the CR condition, 3% and 3.1% were discarded for the right and left hands, respectively. Lastly, in the SCR condition, 7.2% and 5.1% were discarded for the right and left hands, respectively.

### 2.7. Statistical Analysis

Statistical analysis was performed using Matlab 2020b. The normal distribution of the data was verified for all variables with the Shapiro–Wilk test (α = 0.05). Therefore, parametric methods were used. First, we calculated the means and standard deviations for each subject separately so as to represent the variables RT, DMK_TIME_, and EMD_TIME_. These metrics were calculated for each condition and for each hand. Second, the input data for the following statistical analyses were the previously calculated means. Subsequently, we performed three types of tests to assess hypotheses according to the following: (1) for comparisons among conditions, we employed One-way ANOVA with Bonferroni–Holm post-hoc analysis; (2) when comparing between hands—right vs. left—we used the Student t-test for paired samples; and (3) for inter-group comparisons—CON vs. ATH—we applied the Welch t-test for independent samples. The magnitude of difference between comparisons was calculated using Cohen’s d effect size following the criteria indicated by Cohen [31] for the t-tests as trivial (<0.2), small (0.2–0.4), medium (0.4–0.8), and large (>0.8). Any effect size over 0.4 was considered indicative of meaningful differences between groups or hands. Type I error rate was set at the conventional significance level (α = 0.05). Due to the small sample size in the ATH group, we conducted post-hoc power (php) analyses to determine the probability of rejecting a null hypothesis assuming that it is false, as reported for cases where the *p*-value was significant. The type II error rate was estimated through php analyses (β = 0.80).

## 3. Results

Table 1 and Table 2 present a summary of the results for the study variables. The outcomes of the statistical comparisons are detailed in the three following subsections for each variable.

### 3.1. Analysis of Reaction Time (RT)

**Figure 3:**
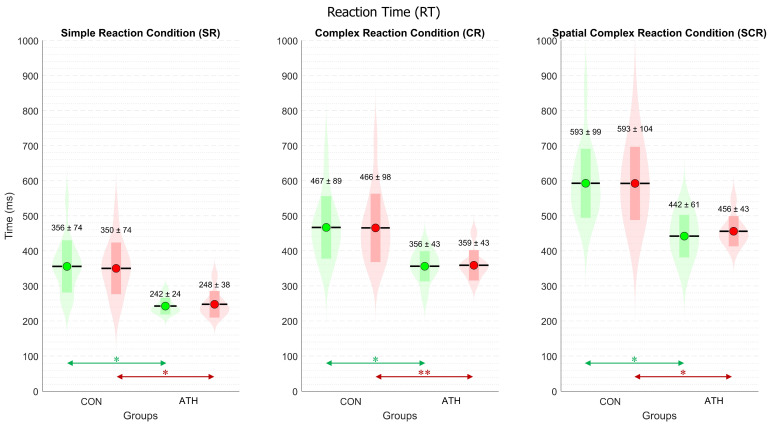
Violin plot of reaction time (RT)**.** Three panels for the three experimental conditions. The control (CON) and athletes (ATH) groups are on the horizontal axis. For the three panels, the following color codes are the same. The green color represents the RT for the right hand, while the red color represents the RT for the left hand. The violin-shaped plot represents the normal distribution of all the variables. The horizontal black lines represent the mean (M), while the colored rectangles are the standard deviation (std). The numerical values are M ± std. * *p* < 0.001 ** *p* < 0.005.

Figure 3 displays the mean ± standard deviation for each group and each hand in the three conditions. Multiple statistical analyses with ANOVA revealed significant differences among all conditions. These differences indicate that the increasing task complexity was reflected in the RT, in line with expectations, for both the right hand of the CON group (F(2,45) = 29.1, *p* < 0.001) and the left hand (F(2,45) = 27.3, *p* < 0.001). The result were similar for the right hand of the ATH group (F(2,21) = 39.4, *p* < 0.001) and the left hand (F(2,21) = 50.3, *p* < 0.001).

For inter-group comparisons, RT showed significant differences in all conditions. For the right hand, SR—t(19) = 5.5, *p* < 0.001, d = 1.8, php = 0.25; CR—t(19) = 4.1, *p* < 0.001, d = 1.43, php = 0.23; SCR—t(20) = 4.6, *p* < 0.001, d = 1.71, php = 0.22, as indicated by green arrows in Figure 3. For the left hand, SR—t(22) = 4.5, *p* < 0.001, d = 1.59, php = 0.23; CR—t(21) = 3.7, *p* < 0.005, d = 1.27, php = 0.24; SCR—t(21) = 4.5, *p* < 0.001, d = 1.52, php = 0.24, as indicated by red arrows in Figure 3. All the effect sizes were large, according to the reported Cohen’s d.

For intra-group comparisons between hands, RT did not reveal significant differences in any case, contrary to expectations, as all participants reported being right-handed.

### 3.2. Analysis of Decision-Making Time (DMK_TIME_)

**Figure 4:**
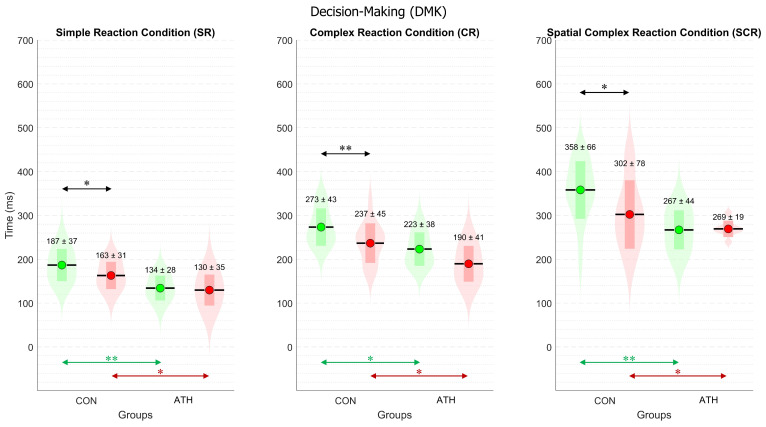
Violin plot of decision-making time (DMK_TIME_)**.** Three panels for the three experimental conditions. The control (CON) and athlete (ATH) groups are on the horizontal axis. For the three panels, the following color codes are the same. The green color represents the DMK_TIME_ for the right hand, while the red color represents the DMK_TIME_ for the left hand. The violin-shaped plot represents the normal distribution of all the variables. The horizontal black lines represent the mean (M), while the colored rectangles are the standard deviation (std). The numerical values are M ± std. * *p* < 0.05, ** *p* < 0.005.

Figure 4 displays the mean ± standard deviation for each group and each hand in the three conditions. Similar to RT, multiple statistical analyses with ANOVA revealed significant differences among all conditions for DMK_TIME_. These differences indicate that the increase in task complexity was reflected in the time required for decision-making, as expected, for both the right hand of the CON group (F(2,45) = 46.9, *p* < 0.001) and the left hand (F(2,45) = 25.5, *p* < 0.001). The result were similar for the right hand of the ATH group (F(2,21) = 25.9, *p* < 0.001) and the left hand (F(2,21) = 36.1, *p* < 0.001).

For inter-group comparisons, DMK_TIME_ showed significant differences in all conditions. For the right hand, SR—t(18) = 3.9, *p* < 0.005, d = 1.53, php = 0.21; CR—t(15) = 2.9, *p* < 0.05, d = 1.22, php = 0.2; SCR—t(19) = 4.0, *p* < 0.001, d = 1.52, php = 0.22, as indicated by green arrows in Figure 4. For the left hand, SR—t(12) = 2.27, *p* < 0.05, d = 1.02, php = 0.18; CR—t(15) = 2.6, *p* < 0.05, d = 1.08, php = 0.2; SCR—t(12) = 2.3, *p* < 0.05, d = 1.02, php = 0.26, as indicated by red arrows in Figure 4. All the effect sizes were large according to the reported Cohen’s d.

For intra-group comparisons between hands, DMK_TIME_ did not reveal significant differences in the ATH group. For the CON group, DMK_TIME_ for the right hand was significantly longer than for the left hand under every condition, such as in SR (t(15) = 2.7, *p* < 0.05, d = 0.67, medium effect size, php = 0.71), in CR (t(15) = 4.5, *p* < 0.001, d = 1.12, large effect size, php = 0.98), and in SCR (t(15) = 2.9, *p* < 0.05, d = 0.72, medium effect size, php = 0.77), all indicated by black arrows in Figure 4.

### 3.3. Analysis of Electromechanical Delay (EMD_TIME_)

**Figure 5:**
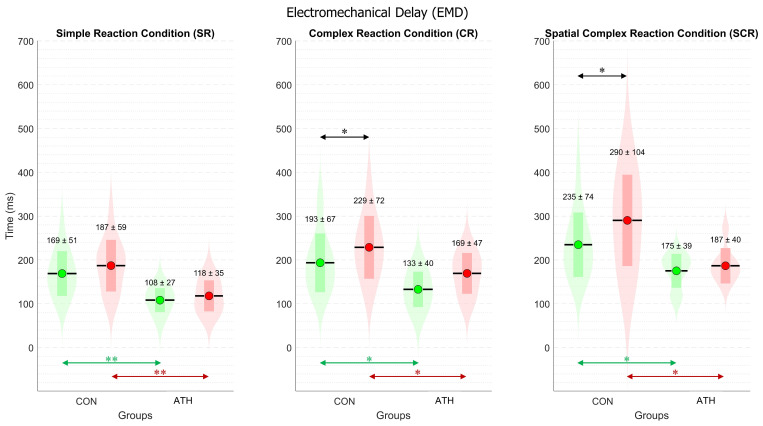
Violin plot of electromechanical delay (EMD_TIME_). Three panels for the three experimental conditions. The control (CON) and athletes (ATH) groups are on the horizontal axis. For the three panels, the following color codes are the same. The green color represents the EMD_TIME_ for the right hand, while the red color represents the EMD_TIME_ for the left hand. The violin-shaped plot represents the normal distribution of all the variables. The horizontal black lines represent the mean (M), while the colored rectangles are the standard deviation (std). The numerical values are M ± std. * *p* < 0.05, ** *p* < 0.005.

Figure 5 displays the mean ± standard deviation for each group and each hand in the three conditions. Statistical analysis with ANOVA revealed non-significant differences among conditions since *p*-values were higher than corrected alpha (α = 0.016) for multiple comparisons.

For inter-group comparisons, EMD_TIME_ showed significant differences in all conditions. For the right hand, SR—t(21) = 3.8, *p* < 0.005, d = 1.35, php = 0.23; CR—t(21) = 2.7, *p* < 0.05, d = 1.01, php = 0.22; SCR—t(21) = 2.6, *p* < 0.05, d = 0.93, php = 0.23, as indicated by green arrows in Figure 5. For the left hand, SR—t(21) = 3.6, *p* < 0.005, d = 1.31, php = 0.23; CR—t(20) = 2.4, *p* < 0.05, d = 0.92, php = 0.22; SCR—t(21) = 2.3, *p* < 0.05, d = 0.91, php = 0.25, as indicated by red arrows in Figure 5. All the effect sizes were large according to the reported Cohen’s d.

For intra-group comparisons between hands, EMD_TIME_ did not reveal significant differences in the ATH group. While for the CON group, significant differences were found, indicating that EMD_TIME_ for the right hand was significantly faster than for the left hand under only two conditions: in CR (t(15) = 2.8, *p* < 0.05, d = 0.71, medium effect size, php = 0.76), and in SCR (t(15) = 2.4, *p* < 0.05, d = 0.62, medium effect size, php = 0.63), as depicted by black arrows in Figure 5.

## 4. Discussion

This study proposes a breakdown of reaction time (RT) using EMG signals for further analysis. By identifying the agonist motor muscle via the detection of initial muscle contraction, RT was divided into two components: decision-making (DMK) and electromechanical delay (EMD). This approach allowed us to asses processing speed in athletes and controls by examining inter-group, inter-conditions, and inter-limb differences.

RT is a relatively easy measure to obtain; the results show that RT increased with task difficulty, according to our expectations and consistent with previous studies [32,33,34]. Our findings reveal that the time elapsed between the onset of a stimulus and the initiation of the response is independent of the hand to be moved, according to the results of multiple comparisons with ANOVA. Given that all participants were right-handed, higher performance in the RT of the dominant hand would have been expected, in line with some previous findings [7,8,17]. However, other studies have suggested that this advantage is not generalizable, as it depends on other factors such as target position compatibility [35,36] or free hand choice [37]. In contrast to the aforementioned studies [35,36] that analyzed performance with the movement phase included, our study used the onset of movement as the end of the RT, and therefore the results are not directly comparable.

The design of the experimental protocol allowed for revealing significant differences between control (CON) and athletes (ATH), regardless of the hand to be moved. In the simple reaction (SR) condition, the CON group had a performance of ~350 ms, while for ATH it was ~250 ms. In the complex (CR) condition, the CON group had an RT of ~460 ms, and for ATH, it was ~360 ms. Finally, in the spatial complex reaction (SCR) condition, the RT was ~600 ms for CON and ~450 ms for ATH. The relationship between both groups revealed a difference of ~30% in favor of athletes, which remained consistent across all three conditions. These results are consistent with previous findings [17,19,20] where athletes demonstrated higher performance in reaction tasks. This advantage may be due to efficiency in the interaction between brain networks during motor processing [38,39], a greater capacity for motor unit recruitment [40,41,42], and reduced reactive inhibition [43,44]. In this sense, our work has focused on accurately determining these differences considering the RT components previously defined.

On one hand, similar to RT, DMK_TIME_ showed significant differences between difficulty conditions according to multiple comparisons analysis with ANOVA. These differences may be attributed to the time required for processes within DMK (i.e., perceptual processes and motor planning). The response in the SR condition may have a greater motor component than perceptual, as the motor goal and motor command were predefined, and participants only awaited the appearance of the stimulus to initiate the response. Meanwhile, the increased difficulty in the CR and SCR conditions may increase the perceptual component, while the motor component is significantly minor, as suggested by Wong et al. [3].

Significant differences were found for DMK_TIME_ between groups, in all conditions and for both hands. As shown in Figure 4, DMK_TIME_ showed significant differences between hands only for the CON group, while the same phenomenon was not observed for ATH. Control participants exhibited DMK_TIME_ for the left hand that was ~20% faster compared to the right hand. This finding was surprising, as it was expected that the right-hand dominance of the participants would generate an advantage in DMK_TIME_. This suggests that control participants may employ different mechanisms for decision-making and motor planning depending on the hand to move [38]. For instance, prior studies in right-handed participants [45,46] showed broad activation across parietal and frontal regions in the left hemisphere during movements for either hand, whereas activation of homologous regions only in the right hemisphere tends to be more limited to left-hand actions. This evidence suggests that the motor planning in control participants differs between hemispheres, as observed in our previous work [25]. This could be explained by the greater inhibitory mechanisms of the left hemisphere for the right hand during movement preparation in right-handers, as suggested by Klein et al. [37]. They showed a corticospinal excitability that was initially increased between the left hemisphere and the right-hand muscle before muscle contraction, and then showed a strong reduction (larger inhibition). This phenomenon was not the same for the right hemisphere and left-hand muscle. This suggests that, at least for right-handers, left-hemisphere motor representations have a lower threshold than right-hemisphere motor representations. If the left hemisphere requires more inhibitory control in right-handers, this might imply an advantage of left-hand processing speed. On the other hand, the absence of right vs. left differences in DMK_TIME_ for the ATH group suggests that these mechanisms of inhibiting right-hand movement could be attenuated.

It was expected that DMK_TIME_ for the right hand would be shorter compared to the left in athletes who extensively use their right arm in daily sport practice. These findings would result in a temporal advantage in processing speed for athletes with asymmetrical use of the left arm. To investigate this further, new experiments using the same protocol with athletes of both handedness are needed. In this context, the present analysis of DMK_TIME_ is proposed. It allows the investigation of such differences.

Lastly, the EMD_TIME_ did not reveal significant differences between difficulty conditions according to multiple comparisons analysis. This evidence suggests that RT variability is primarily influenced by DMK_TIME_, which did show substantial differences between conditions, as analyzed above. Controls showed shorter EMD_TIME_ for the right arm compared to the left. It is important to acknowledge that this difference was only observed in CR and SCR conditions. This observation might suggest a potential influence of daily right-hand use, albeit not conclusively demonstrated as a significant difference. On the other hand, in the ATH group, no differences between limbs were observed. This finding again surprises, as it was expected that EMD_TIME_ for the right arm would be shorter compared to the left due to the predominant use of the muscles in the right arm during respective sports practices. A possible explanation for the lack of difference could be a greater involvement of synergistic muscles in left-arm movement preparation [47,48], resulting in shorter RTs. However, this type of analysis was not the aim of the study. Functional connectivity analyses are needed to study the interaction between muscles, which would allow the quantification of muscle synergy participation and its relationship with cortical processes [49,50].

The present study revealed that focusing on DMK time assessment could be an innovative approach for evaluating disparities rather than RT. Moreover, the present proposal might be applied for tracking adaptations during physical–motor training. For instance, Theofilou et al. [14] demonstrated that a visual training program involving simple and cognitive tasks may improve RT. However, RT was assessed without differentiation between the DMK and EMD components. Our approach could delve deeper into identifying whether the improvements observed can be attributed to the central or peripheral level. This would allow for optimizing the design of training activities based on the expected adaptations. Improvements in the DMK could result from implementing motor–cognitive tasks. While improvements in the EMD component could be associated with simple response tasks, they may be attributed to neuromuscular adaptations, such as the increased firing frequency and synchronization of motor units [51,52]. Additionally, this approach could be useful in monitoring the effects of musculoskeletal injuries by assessing RT, as suggested by previous studies [53,54,55]. In this line, the present proposal of breaking down RT could help with researching into mechanisms of sensorimotor processing alterations in participants with musculoskeletal injuries.

Finally, it is important to consider some potential limitations of our study. The small and imbalanced sample size limits the generalizability of the results, and the post-hoc power analysis results reported were predominantly low (<0.8). However, it is important to note that the effect sizes were predominantly large (>0.8) or medium (0.4–0.8). Another limitation was the absence of left-handed participants in both groups, hindering the comparisons with more powerful statistical analysis. Additionally, handedness was assessed by asking controls which hand they used to write, as the intention was to establish which arm had greater daily usage. Similarly, the athletes were asked about which hand they use in their sports practice. However, specific evaluations of dominance were not conducted. Future studies should consider precise assessments to discriminate between preference and dominance. Lastly, considering all athletes were recruited from different sports, caution should be exercised, and further studies are necessary to validate whether these findings can be extrapolated to different sports, to different experience levels, and to the age-related impact.

## 5. Conclusions

This study highlights the importance of considering decision-making (DMK) and electromechanical delay (EMD) for assessing processing speed in motor reaction tasks. We found that as task complexity increased, reaction time (RT) differences were primarily driven by variations in DMK time rather than EMD. The results suggest that DMK time may offer higher sensitivity in evaluating processing speed compared to RT.

The practical implications of this study can provide knowledge for optimizing training methods, specifically when reaction is essential to improving performance. The type and characteristics of the practiced sport determine which component of RT could be more relevant to assess. This proposal for identifying components of RT would enable coaches to monitor whether the training programs applied to athletes induce adaptations in motor–cognitive processing (central level) or neuromuscular processes (peripheral level). On the other hand, the theoretical implications of these findings could be relevant to delving deeper into processing speed research. Finally, this study could have implications for our understanding of handedness by exploring the underlying mechanisms of processing speed, which could be better represented through decision-making time.

## Figures and Tables

**Table 1:** Control Group (CON). Time expressed in milliseconds as mean ± standard deviation.

	Right (Dominant)			Left (Non-Dominant)		
Conditions	RT	DMK_TIME_	EMD_TIME_	RT	DMK_TIME_	EMD_TIME_
SR	356 ± 74	187 ± 37	169 ± 51	350 ± 74	163 ± 31	187 ± 59
CR	467 ± 89	273 ± 43	193 ± 67	466 ± 98	237 ± 45	229 ± 72
SCR	593 ± 99	358 ± 66	235 ± 74	593 ± 104	302 ± 78	290 ± 104

Simple Reaction (SR), Complex Reaction (CR), Spatial Complex Reaction (SCR), reaction time (RT), decision-making (DMK), electromechanical delay (EMD).

**Table 2:** Athletes Group (ATH)**.** Time expressed in milliseconds as mean ± standard deviation.

	Right (Dominant)			Left (Non-Dominant)		
Conditions	RT	DMK_TIME_	EMD_TIME_	RT	DMK_TIME_	EMD_TIME_
SR	242 ± 24	134 ± 28	108 ± 27	248 ± 38	130 ± 35	118 ± 35
CR	356 ± 43	223 ± 38	133 ± 40	359 ± 43	190 ± 41	169 ± 47
SCR	442 ± 61	267 ± 44	175 ± 39	456 ± 43	269 ± 19	187 ± 40

Simple Reaction (SR), Complex Reaction (CR), Spatial Complex Reaction (SCR), reaction time (RT), decision-making (DMK), electromechanical delay (EMD).

## Data Availability

The data presented in this study are available upon request from the corresponding author. The data are not publicly available due to data protection policies practiced at our institution, as they contain information that could compromise the privacy of research participants.

## Supplementary Materials

The following supporting information can be downloaded at: https://www.mdpi.com/article/10.3390/sports12060151/s1. Figure S1: Performance across repetitions under the simple reaction (SR) condition; Figure S2: Performance across repetitions under the complex reaction (SR) condition; Figure S3: Performance across repetition under the spatial complex reaction (SCR) condition. Table S1: Principal outcomes from linear regression models pooled by group and conditions.
