# Supplementary material for: Decision-Making Time Analysis for Assessing Processing Speed in Athletes during Motor Reaction Tasks
Source: Sports (Basel). 2024 May 29;12(6):151. doi: 10.3390/sports12060151 (PMC11207928; doi:10.3390/sports12060151)
Supplement: Supplementary file 1 [file sports-12-00151-s001.zip › sports-2963267-supplementary.pdf]

SUPPLEMENTARY MATERIAL

Article title:

Decision-Making Time Analysis for Assessing Processing Speed in Athletes during Motor Reaction Tasks

Authors:

Leonardo Ariel Cano, Gonzalo Daniel Gerez, María Soledad García, Ana Lía Albarracín, Fernando Daniel Farfán, and Eduardo Fernández-Jover

Control of potential Learning Effect:

The potential learning effect related to a non-randomized order for presenting the conditions was evaluated prior the study. Several pilot tests were carried out, which allowed us to determine that presenting the conditions in a constant order across participants was beneficial for three reasons. Firstly, we found that following a difficulty-based incremental sequence facilitated participants' quick adaptation to the tasks, compared to a random order that made it challenging to grasp the instructions and affected responses in several trials. Secondly, we observed under a random presentation of conditions, the baseline/preparation state could be very different between control subjects and athletes. With the proposed protocol, we minimize this bias in the response. Finally, we observed that despite the progressive difficulty presentation, the both times -Reaction Time (RT) and Decision-Making (DMK) time- maintained an incremental trend according to difficulty, as also reflected in the results presented in this work. Therefore, a non-randomized presentation of conditions did not imply a significant learning effect that could influence hypothesis testing.

Additionally, the presence/absence of learning effects within each condition was assessed by analyzing each participant's responses across repetitions. We used parametric linear regression method to test a systematic trend across repetitions. No systematic decrease in the variables (i.e. RT, DMK, and EMD) was observed from the first to the last repetition in any of the experimental conditions. This lack of systematic change in performance throughout repetitions confirmed the absence of a learning effect in the study. The Table S1 presents the principal models' outcomes: determination coefficient (R^2), p-value, and the regression coefficient (slope). In this sense, R^2 near to zero with p-value over than 0.05 indicates non-significant trend. If p-value is below than 0.05, the slope indicates a systematic increase/decrease depending on the plus/minus symbol, respectively. In this study, no systematic decreasing trend with p-value <0.05 was observed.

The Figures S1, S2, and S3 show the Reaction Time (RT) during the three difficulty conditions: Simple Reaction (SR), Complex Reaction (CR), and Spatial Complex Reaction (SCR). Every figure present a left panel depicting the performance of the Control group (CON), while the right panel comprises the Athletes group (ATH).

Table S1. Principal outcomes from Linear Regression Models pooled by group and conditions

Simple Reaction (SR) condition

	Control Group (CON)						Athletes Group (ATH)					
	Right (dominant)			Left (non-dominant)			Right (dominant)			Left (non-dominant)		
	R^2	p-value	slope	R2	p-value	slope	R^2	p-value	slope	R2	p-value	slope
RT	0.014	0.618	-0.482	0.071	0.255	-0.137	0.057	0.317	-0.411	0.005	0.767	-0.129
DMK	0.102	0.168	1.289	<0.001	0.921	-0.048	0.023	0.523	0.540	0.032	0.446	-0.393
EMD	0.072	0.252	-0.982	0.144	0.098	-1.959	0.191	0.053	1.310	0.005	0.754	-0.189

Complex Reaction (CR) condition

	Control Group (CON)						Athletes Group (ATH)					
	Right (dominant)			Left (non-dominant)			Right (dominant)			Left (non-dominant)		
	R^2	p-value	slope	R2	p-value	slope	R^2	p-value	slope	R2	p-value	slope
RT	0.058	0.350	0.960	0.329	0.01	2.783	0.015	0.600	-0.479	0.009	0.685	.0379
DMK	0.126	0.161	2.198	0.017	0.613	-2.372	0.027	0.483	-0.612	0.110	0.152	1.937
EMD	0.033	0.479	-1.237	0.089	0.244	5.156	0.001	0.884	0.132	0.106	0.160	-1.557

Spatial Complex Reaction (SCR) Condition

	Control Group (CON)						Athletes Group (ATH)					
	Right (dominant)			Left (non-dominant)			Right (dominant)			Left (non-dominant)		
	R^2	p-value	slope	R2	p-value	slope	R^2	p-value	slope	R2	p-value	slope
RT	0.105	0.163	-1.850	0.046	0.442	-1.955	0.049	0.343	1.078	0.033	0.442	0.618
DMK	0.015	0.599	-1.330	0.033	0.516	1.790	0.007	0.725	-0.701	<0.001	0.983	0.038
EMD	0.003	0.797	-0.519	0.106	0.234	-3.745	0.042	0.383	1.7793	0.006	0.739	0.579

Reaction Time (RT), Decision-making (DMK), Electromechanical delay (EMD), Determination coefficient (R^2),

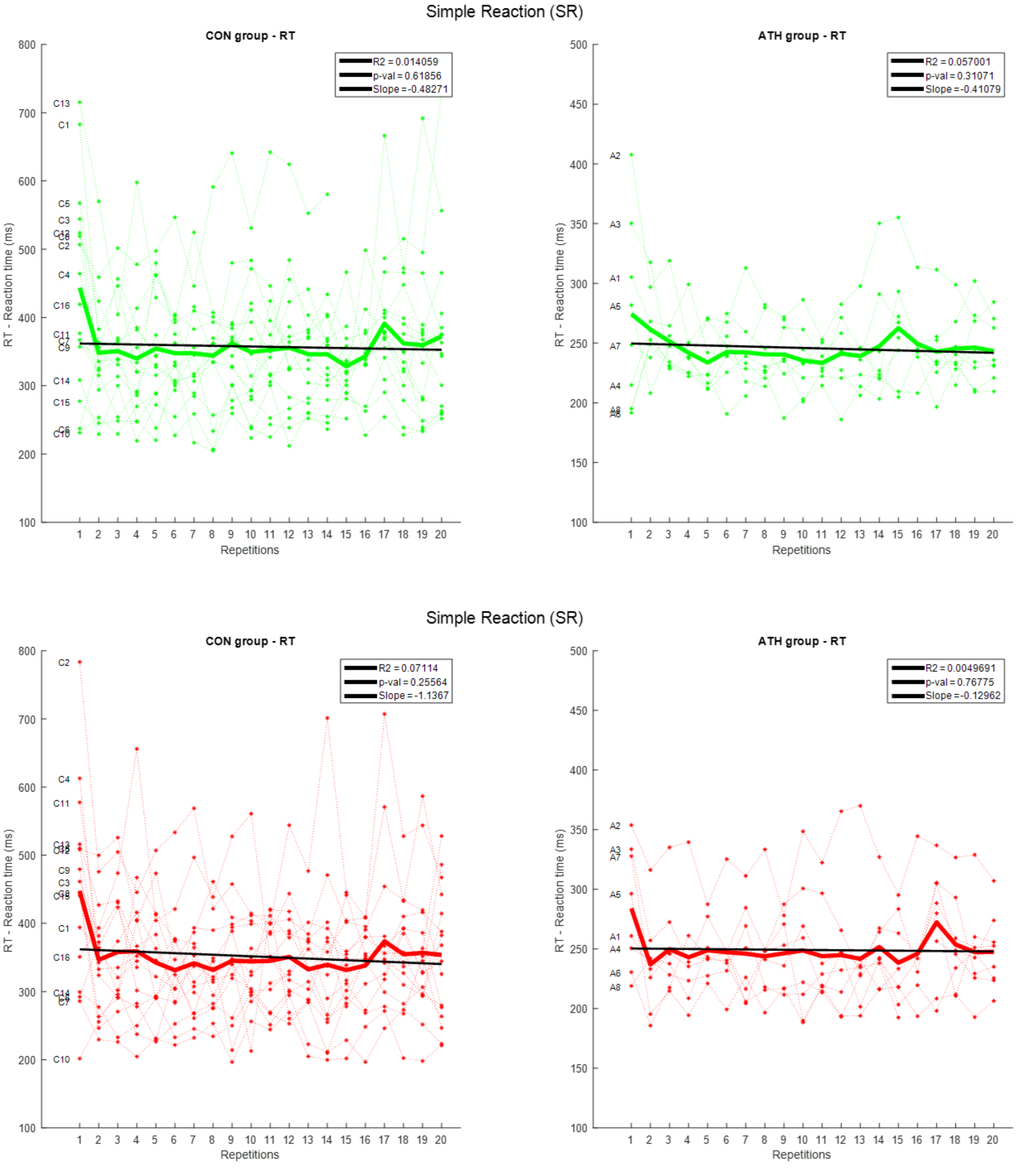

Figure S1. Performance across repetitions during Simple Reaction (SR) condition. On y-axis, Reaction Time (RT) in milliseconds. On x-axis, numbered repetitions. On the left column, the Control group (CON). On the right column, the Athletes group (ATH). The upper row presents the right-hand performance, colored with green. The lower row presents the left-hand performance, colored with red. The dotted (·) lines depict participants' performance across condition, participants are identified by codes (C for controls, A for athletes), the circles (•) represents the RT value for each repetition. The wide lines colored with green/red represent the averaged data for each repetition. The wide line colored with black is the linear model. The model's outcomes are presented in each panel.

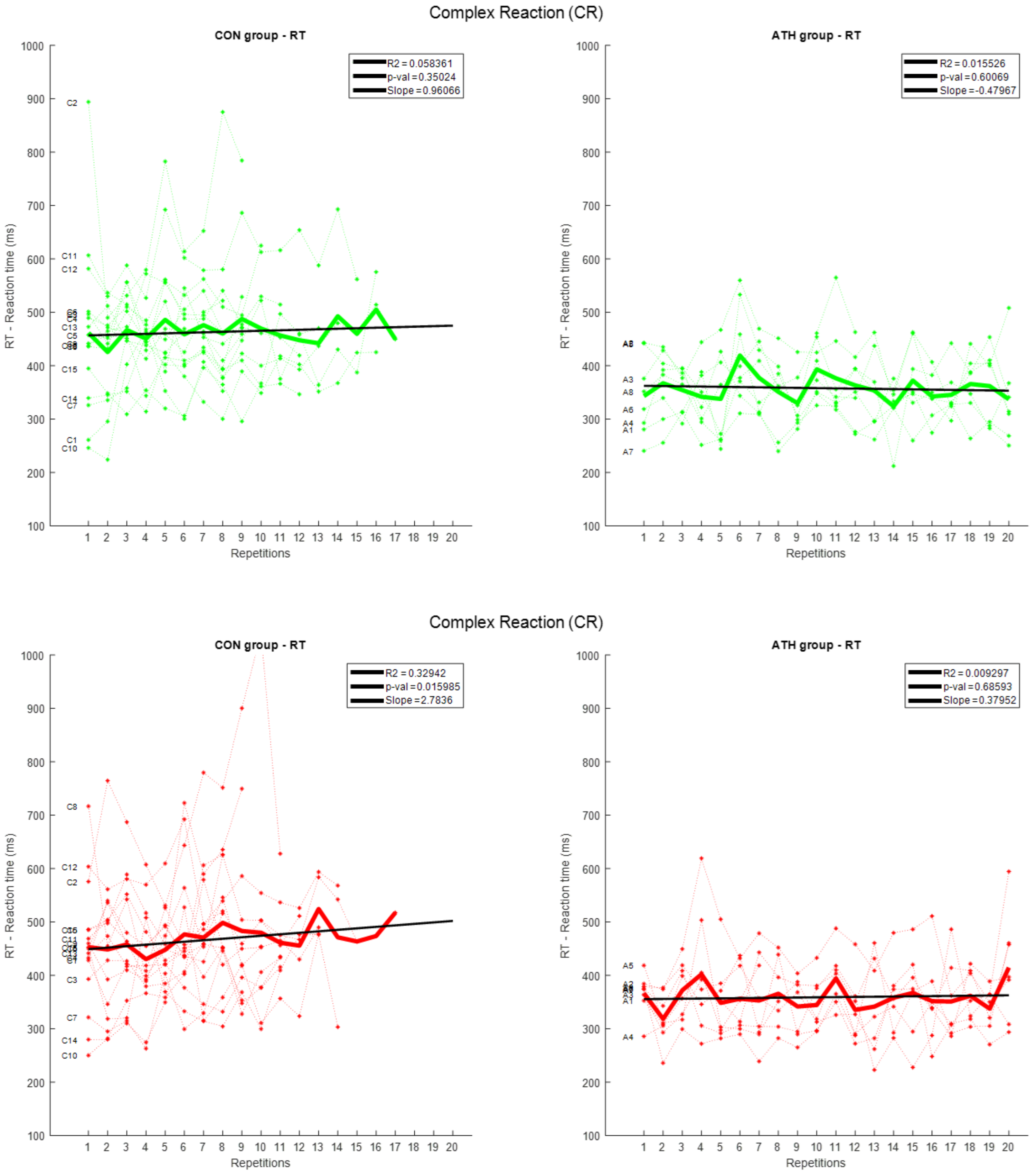

Figure S2. Performance across repetitions during Complex Reaction (SR) condition. On y-axis, Reaction Time (RT) in milliseconds. On x-axis, numbered repetitions. On the left column, the Control group (CON). On the right column, the Athletes group (ATH). The upper row presents the right-hand performance, colored with green. The lower row presents the left-hand performance, colored with red. The dotted (·) lines depict participants' performance across condition, participants are identified by codes (C for controls, A for athletes), the circles (•) represents the RT value for each repetition. The wide lines colored with green/red represent the averaged data for each repetition. The wide line colored with black is the linear model. The model's outcomes are presented in each panel.

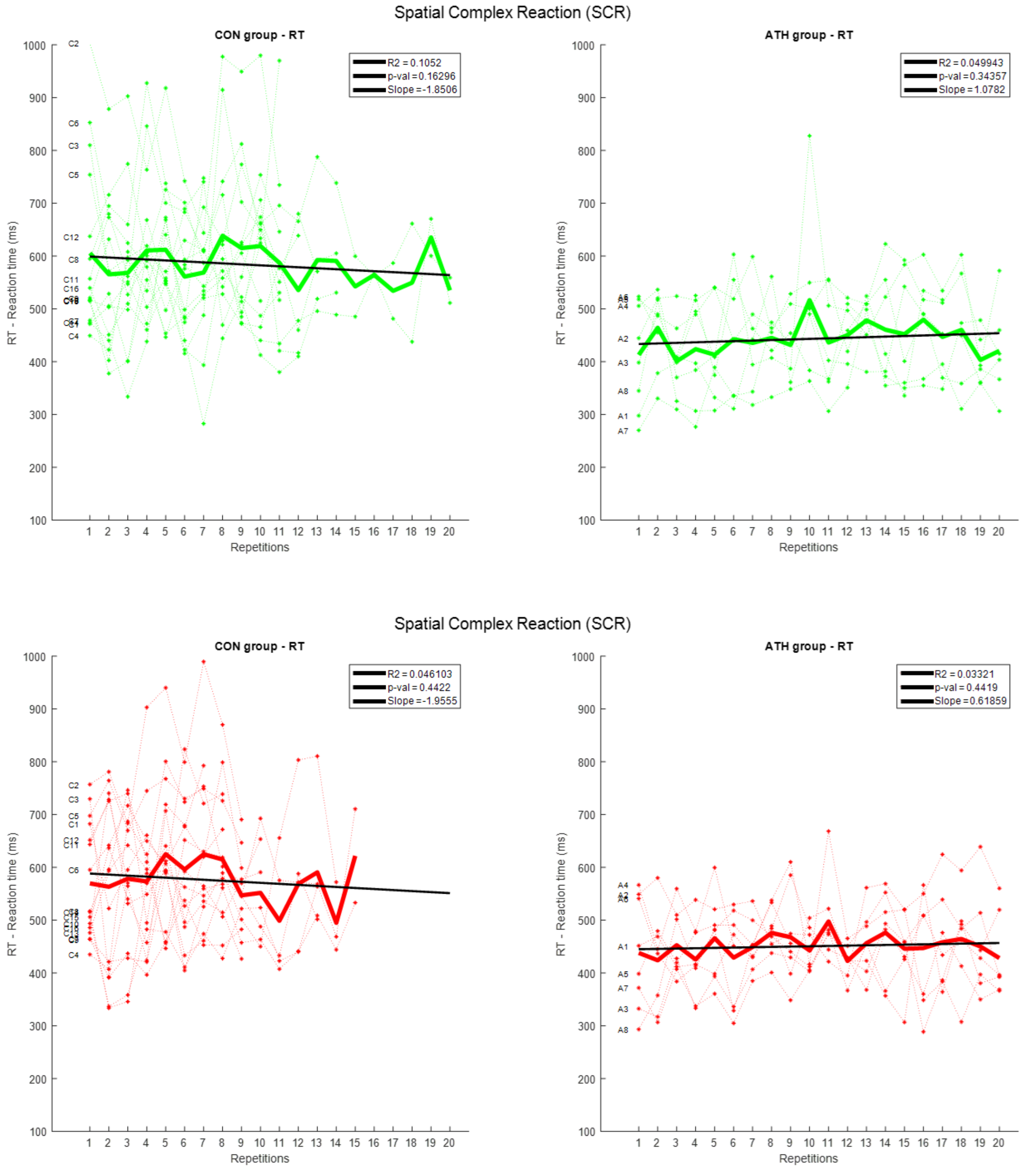

Figure S3. Performance across repetition during Spatial Complex Reaction (SCR) condition. On y-axis, Reaction Time (RT) in milliseconds. On x-axis, numbered repetitions. On the left column, the Control group (CON). On the right column, the Athletes group (ATH). The upper row presents the right-hand performance, colored with green. The lower row presents the left-hand performance, colored with red. The dotted (·) lines depict participants' performance across condition, participants are identified by codes (C for controls, A for athletes), the circles (•) represents the RT value for each repetition. The wide lines colored with green/red represent the averaged data for each repetition. The wide line colored with black is the linear model. The model's outcomes are presented in each panel.
